# MULTISENSORY IMPAIRMENTS AND BRAIN VOLUMES IN MID-TO-LATE LIFE

**DOI:** 10.1093/geroni/igac059.2531

**Published:** 2022-12-20

**Authors:** Amal Wanigatunga, Chenxin Tian, Yang An, Yurun Cai, Alden Gross, Eleanor Simonsick, Jennifer Schrack, Yuri Agrawal

**Affiliations:** Johns Hopkins Bloomberg School of Public Health, Baltimore, Maryland, United States; Johns Hopkins Bloomberg School of Public Health, Baltimore, Maryland, United States; National Institute on Aging, Baltimore, Maryland, United States; University of Pittsburgh, Pittsburgh, Pennsylvania, United States; Johns Hopkins Bloomberg School of Public Health, Baltimore, Maryland, United States; National Institute on Aging, Baltimore, Maryland, United States; Johns Hopkins Bloomberg School of Public Health, Baltimore, Maryland, United States; Johns Hopkins School of Medicine, Baltimore, Maryland, United States

## Abstract

Age-related sensory impairment, such as loss in vision or hearing, have been linked to poor brain health. Yet, the relationship between co-occurring sensory impairments and brain volumes remains unclear. We used cross-sectional sensory and brain imaging data from 208 cognitively normal participants of the Baltimore Longitudinal Study of Aging (mean age 72 years; 59% women). Sensory impairments were separately identified with vision, hearing, smell, proprioception, and vestibular function testing. Brain imaging volumes were derived using an automated multi-atlas approach. Multiple linear regression models were used to estimate brain volumetric differences by number of sensory impairments (0-5) or by multisensory impairment status (MSI; ≥2 impairments). For every one additional sensory impairment, there was lower volume in the orbitofrontal gyrus (beta=0.35 cc [SE=0.17], p=0.04) but higher volumes in the caudate (0.14 cc [0.05], p=0.006) and putamen (0.13 cc [0.06], p=0.043). Participants with MSI (versus no MSI) had lower volumes in superior frontal gyrus (-1.01 cc [=0.34], p=0.003), lower orbitofrontal gyrus (-0.91 cc [0.38], p=0.018), superior parietal lobe (0.68 cc [0.27], p=0.011), and precuneus (-0.74 cc [0.35], p=0.038), but similar volumes in the caudate (p=0.12) and putamen (p=0.14). A negative relationship between MSI and brain volume across multiple regions was largely observed yet our exploratory findings raise the possibility that MSI is associated with compensatory maintenance of brain volume structures in the basal ganglia. Future directions include replication of these findings in other studies and longitudinal analyses to determine how MSI relates to brain atrophy.

